# Supplementary material for: Effects of Magnesium on Transcriptome and Physicochemical Index of Tea Leaves
Source: Plants (Basel). 2023 Apr 28;12(9):1810. doi: 10.3390/plants12091810 (PMC10181054; doi:10.3390/plants12091810)
Supplement: Supplementary file 1 [file plants-12-01810-s001.zip › plants-2314408-supplementary.pdf]

Supplementary Materials

Table S1 Overview of the tea leaves metagenomic sequencing

Sample	Raw Reads	Clean Reads	Clean Base(G)	Error Rate(%)
M1-1	52880364	52001448	7.80	0.03
M1-2	58706344	57655662	8.65	0.03
M1-3	59114616	58008120	8.70	0.03
M2-1	55994064	54941134	8.24	0.03
M2-2	41541042	40792498	6.12	0.03
M2-3	47635590	46977510	7.05	0.03
M3-1	47603320	46802894	7.02	0.03
M3-2	47587670	46660894	7.00	0.03
M3-3	43976934	42789208	6.42	0.03
Total/Average	455039944	446629368	67.00	0.03

Note: M1: Control; M2: The Mg concentration is 0.4m mol/L; M3: The Mg concentration is 0.8 mmol/L

Table S2 Efficiency statistics of transcriptome data compared with reference genomes

Sample	Total Reads	Reads mapped	Unique mapped	Multi mapped	Read1 mapped	Read2 mapped	'+' mapped	'-' mapped
M1-1	46802894	41082667(87.78%)	38496896(82.25%)	2585771(5.52%)	19424164(41.50%)	19072732(40.75%)	19267507(41.17%)	19229389(41.09%)
M1-2	46660894	40138661(86.02%)	37624398(80.63%)	2514263(5.39%)	19000175(40.72%)	18624223(39.91%)	18831651(40.36%)	18792747(40.28%)
M1-3	42789208	37701908(88.11%)	35349290(82.61%)	2352618(5.50%)	17766723(41.52%)	17582567(41.09%)	17693883(41.35%)	17655407(41.26%)
M2-1	52001448	45341865(87.19%)	42480283(81.69%)	2861582(5.50%)	21415440(41.18%)	21064843(40.51%)	21255910(40.88%)	21224373(40.81%)
M2-2	57655662	50225362(87.11%)	47036125(81.58%)	3189237(5.53%)	23787066(41.26%)	23249059(40.32%)	23534996(40.82%)	23501129(40.76%)
M2-3	58008120	50528574(87.11%)	47346941(81.62%)	3181633(5.48%)	23890368(41.18%)	23456573(40.44%)	23689649(40.84%)	23657292(40.78%)
M3-1	54941134	49168624(89.49%)	46164824(84.03%)	3003800(5.47%)	23162596(42.16%)	23002228(41.87%)	23108951(42.06%)	23055873(41.96%)
M3-2	40792498	36168709(88.67%)	33998852(83.35%)	2169857(5.32%)	17136903(42.01%)	16861949(41.34%)	17020649(41.72%)	16978203(41.62%)
M3-3	46977510	42034975(89.48%)	39542729(84.17%)	2492246(5.31%)	19830648(42.21%)	19712081(41.96%)	19796912(42.14%)	19745817(42.03%)

Note: M1: Control; M2: The Mg concentration is 0.4 mmol/L; M3: The Mg concentration is 0.8 mmol/L; The reference genome was GCF_004153795.1_AHAU_CSS_1_genomic.fna.gz. Download address: https://ftp.ncbi.nlm.nih.gov/genomes/all/GCF/004/153/795/GCF_004153795.1_AHAU_CSS_1/; Total Reads: Total number of clean reads; Reads mapped: The number of reads compared to the reference genome; Unique mapped: The number of reads only compared to the reference genome; Multi mapped: The number of reads multiple compared to the reference genome; Read1 mapped: Number of successes comparison in read1; Read2 mapped: Number of successes comparison in read2; '+' mapped: Number of reads successfully comparison on the positive chain of the genome; '-' mapped: Number of reads successfully comparison on the negative chain of the genome.
